# Ophthalmology

**Published:** 1900-03

**Authors:** F. R. Cross


### OPHTHALMOLOGY.

**Myopia** is an error of refraction, due to elongation of the eyeball, and its conditions vary with its degree. When it does not exceed four or five dioptres it is of little disadvantage, except to those who wish to enter the public services, or whose occupations require particularly good far vision; but as the amount increases, it causes more and more damage to the sight, until in its high degrees it becomes a very serious disease. Infants are almost always hypermetropic, and the higher grades of myopia are reached in every instance by gradual development through the lower, so that any case, however slight, may be on its way to the highest degree of the defect. It is therefore of the greatest importance that thoroughly competent medical advice should be sought in every case of commencing short sight, in order that the disease may be arrested if possible, or at any rate prevented from reaching the extreme amount which would result if all prophylactic measures were disregarded. The problem of defective eyesight can under no conditions be properly dealt with merely by testing the refraction and prescribing spectacles, and there is no member of a family whose future comfort and usefulness will depend more on well-timed medical advice and hygienic treatment than the child who is developing myopia.

An adult who wishes to improve his vision by the use of lenses may do so with the aid of the optician, for his own convenience and at his own risk, but the parent of a child who has "something wrong with the sight" should surely obtain a good medical opinion with regard to the defect and its treatment, as he would do in the case of any other disease. Most opticians seem to consider that nearly all forms of defective sight can be remedied by spectacles, provided that moderately good vision, as shown by the routine methods of testing, can be obtained with their aid. Further, some of them seem to be ill-judged enough to suppose that they are quite competent to advise upon and properly treat cases of short sight, as may be seen from the following paragraph which appears in a publication circulating among opticians and devoted to their interests:—"Should this statement prove to be justified by the results, there may after all be some groundwork for the assertion that myopia above seven dioptres should receive the attention of an ophthalmic surgeon." This comment is made after an abstract of a paper by the writer on "The Operative Treatment of High Myopia," a purely surgical subject. It is a new departure for an optician to be the critic of the work or deductions of an ophthalmic surgeon, and I think the journal in question would be better employed in aiming to improve the construction and fitting of spectacles (a business by no means practically perfect), rather than in quoting scientific papers from medical journals, and that without always giving a proper acknowledgment of the source from which they are taken.

Should the ophthalmic surgeon be consulted only after short sight has developed to a serious degree, his opportunities of benefiting myopic patients will be very distinctly curtailed. Myopes of seven dioptres have eyes which are often permanently damaged: none of them see anything quite distinctly at a distance of more than six inches, while even with the best possible lenses the average vision is reduced to $\frac{6}{12}$; they also require spectacles for reading, and although many of them are able to use their distance glasses for all purposes, others cannot do so, and are put to the trouble and inconvenience of having two pairs.

Moreover, the amount of vision is not by any means a safe guide to the condition of the eye. Thus in two eyes with an equal amount of short sight, a defect of vision in one might result from choroiditis in the neighbourhood of the macula lutea which would call for prolonged and judicious medical treatment, while a more pronounced defect in the other might be accounted for by a congenital faulty development of the nerves, which cannot be cured and need not cause further anxiety.

It is, however, in its highest degrees that short sight causes the greatest inconvenience to patients and damage to their eyes. When the amount of myopia present exceeds thirteen dioptres, the patient is not only unable to see clearly without glasses either at a distance or for reading, but with full correction his best far vision is often reduced to one-fourth of the normal, and he is unable to utilise even this modified sight to its full extent because the glasses cause so much annoyance from prismatic effects and diminution in the apparent size of objects that he usually prefers to wear weaker lenses, in which he is more comfortable although he sees less.

In addition to these defects of vision, highly myopic eyes are especially liable to pathological changes which still further impair their usefulness. The choroid and retina, especially in the neighbourhood of the optic disc and macula lutea, become atrophied, and hemorrhages in the latter situation are of frequent occurrence, leading to loss of central vision. The vitreous becomes fluid and opacities develop in it, giving rise to muscæ and mistiness of sight. Finally, these eyes, owing to stretching of the sclera and degeneration of the vitreous, are peculiarly liable to detachment of the retina. When short sight has reached this extreme degree, it not only interferes with a patient's comfort and capacity to earn his living, but even threatens him with practical blindness, so that we cannot question the wisdom of resorting to heroic measures for its cure, provided that they hold out a good prospect of real benefit.

During the past year special attention has been drawn to the **operative treatment** of high myopia by debates on the subject, which have been held in the Ophthalmological Society of the United Kingdom[1] and in the Société Française d'Ophtalmologie.[2] So long ago as 1765, a Frenchman, the Abbé Desmonceaux, proposed that short sight of high degree should be improved or cured by removal of the crystalline lens. The risk, however, of interfering with eyes which possessed fairly useful vision appeared to most oculists to be so great as to render the proceeding unjustifiable, and it was not until Fukala read a paper on the subject in Vienna in 1889, giving the result of sixteen such operations, and at the same time showed two of his patients, that the procedure began to be adopted experimentally by a considerable number of ophthalmic surgeons. In the ten years which have since elapsed, a sufficient number of cases have been recorded to enable us to form an opinion as to the value of this method of treatment, and it can safely be stated that it has gained a permanent place in eye surgery. The effect of the operation is to simplify the eye as an optical instrument and to diminish its refractive power to a marked degree. Authorities concur in advising that, as a rule, the crystalline lens should not be removed for the cure of a myopia of less than sixteen dioptres, and it has been found by experience that an eye of this focus will, after operation, see best for distance with a lens of approximately +3D, while an eye with a myopia of twenty-two dioptres will when rendered aphakic become practically emmetropic.

The chief benefit derived by patients who have had their myopia cured by operation is that they are able to see distant objects much better, either without glasses or with glasses of weak focus, than they previously could even with the very strong concave lenses which they then required. For purposes of close sight, however, the results of the operation are less satisfactory, as the patient always requires convex glasses unless the original myopia reached approximately thirty dioptres, a degree practically unknown. As a rule, the lens required for reading is (as in the case of patients who have undergone ordinary cataract extraction) one of +4D, in addition to that which gives the best distance vision, but frequently much stronger glasses than this are preferred, for a time at any rate.

In addition to the improvement in vision, a distinct alteration in the appearance of the fundus has been noticed after operation. The thinning of the choroid and the abnormal distance between its vessels so characteristic of progressively myopic eyes become less marked, and the edge of the posterior staphyloma becomes more clear-cut and distinct. These changes would seem to indicate that the operation has not only an optical action, but also a beneficial influence upon the nutrition and tissues of these faulty eyes.

The chief objections to the treatment are the immediate

[1] *Tr. Ophth. Soc. U. Kingdom*, 1899, xix. 160.

[2] *Rev. gén. d'Opht.*, 1899, xviii. 231.

risks from septic infection and glaucoma. The former of these should be avoided by strict asepsis and skilful manipulation, while increased tension can be relieved by evacuation of the swollen lens-substance which causes it. At a later stage detachment of the retina may result, but so far as our present experience has gone, this very serious complication, which is already predisposed to by high myopia, has been found to occur only in a small percentage of cases.

The eye is usually rendered aphakic by discission of the lens. In children one or two needlings may suffice, but in young adults curette-evacuation will also be necessary to avoid glaucoma. In older patients cure by discission is impracticable, and some modified form of extraction of the lens is indicated.

The preparations most commonly used for the treatment of the various forms of **conjunctivitis** are the astringent metallic salts, and of these the most valuable is undoubtedly nitrate of silver. Applications of this drug are decidedly painful and do not penetrate deeply into the tissues, as its acid causes coagulation in the superficial layers of the mucous membrane. In addition to its caustic action, the salt possesses bactericidal qualities which are said to depend upon the metal, of which it contains 63.5 per cent. In the hope of finding a substance which, while free from the undesirable effects of the inorganic salt, would retain its antiseptic properties, experiments have of late years been made with various organic salts of silver. The chief of these are: Argonin, containing 4.25 per cent. of silver; Argentamin, containing 6.35 per cent. of silver; Protargol, containing 8.3 per cent. of silver; Largin, containing 11.1 per cent. of silver.

Protargol and largin seem to be the best, and may with advantage be compared with each other. The former is an albuminate of silver and occurs as a smooth, yellowish powder, which is readily soluble in water (50 to 70 per cent.), and is not precipitated from aqueous solutions by albuminous substances, acids or alkalies. It is not reduced by light, and is unaffected by mixing with cocaine, atropine, or eserine. Its power of penetration is said to be five times as great as that of nitrate of silver.

Largin is a greyish powder, produced by the action of an ammoniacal solution of oxide of silver on an alcoholic solution of the dry product of decomposition of the paranucleoproteids.[1] It contains rather a larger percentage of silver than protargol, but its solubility in water is much less (11.5 per cent.). It would seem that when solutions containing equal percentages of the two drugs are employed, the action of largin should be more powerful than that of protargol, but the greater

[1] *Wien. med. Presse*, 1898, xxxix. 1305; abstract in *Brit. M. J.*, 1898, ii. Epitome, p. 80.

solubility of the latter renders it preferable for use where a very energetic effect is desired. Darier[1] claims to have obtained good results without causing undue irritation with a 33.3 per cent. solution of protargol, which would contain three times as much silver as the saturated solution of largin or as the 2 per cent. solution of nitrate of silver commonly used for brushing the lids.

The argyrosis or brown colouration of the conjunctiva which is well known to follow the incautious use of nitrate of silver has already been observed to occur in a case treated with protargol, and it will probably also be met with as a result of applications of other members of this group of remedies as our experience becomes more extensive.

There seems reason to believe that the claims made on behalf of these preparations will be substantiated. In my own experience they have proved of considerable value, and they certainly cause less inconvenience than solutions of nitrate of silver, but I have found applications of a 20 per cent. solution of protargol give rise to a considerable amount of irritation.

F. R. Cross.

---

